# The effect of transcranial direct current stimulation combined with working memory training on working memory deficits in schizophrenic patients: study protocol for a randomized controlled trial

**DOI:** 10.1186/s13063-022-06776-x

**Published:** 2022-09-30

**Authors:** Xiaolin Zhu, Chao Huang, Hongzhen Fan, Fengmei Fan, Yanli Zhao, Meihong Xiu, Yunhui Wang, Yajun Li, Yunlong Tan, Zhiren Wang, Shuping Tan

**Affiliations:** 1 Beijing Huilongguan Hospital, Peking University Huilongguan Clinical Medical School, Beijing, 100096 People’s Republic of China; 2 Pinghu Branch, Chongqing Three Gorges Central Hospital, Chongqing, 404000 People’s Republic of China

**Keywords:** Cognitive deficits, Transcranial direct current stimulation, Schizophrenia, Working memory training, Randomized controlled trial

## Abstract

**Background:**

Working memory deficits are one of the core and most characteristic clinical features of cognitive impairment in schizophrenia. Cognitive training can improve the cognitive function of patients with schizophrenia. However, the overall and transfer effects of working memory treatment (WMT) require improvement. Numerous studies have confirmed that transcranial direct current stimulation (tDCS) enhances neuroplasticity in the brain, providing a new treatment approach for cognitive impairment in patients with schizophrenia. We hypothesize that a training mode combining “preheating” (tDCS, which changes the neural activity of working memory-related brain regions) and “ironing” (WMT) affords greater cognitive improvements than WMT alone. In addition, this study aims to examine the mechanisms underlying the superiority of tDCS combined with WMT in improving cognitive function in patients with schizophrenia.

**Methods:**

This study will include 120 patients with schizophrenia aged 18–60 years. The patients will be randomized into four groups: the study group (tDCS + WMT), tDCS group (tDCS + simple response training, SRT), WMT group (sham tDCS + WMT), and control group (sham tDCS + SRT). Patients will receive 20-min, 2 mA sessions of active or sham tDCS twice a day on weekdays for 2 weeks. Each stimulation will be immediately followed by a 1 − 2-min rest and 40 min of WMT or SRT. The primary outcome is cognitive function, measured using Repeatable Battery for the Assessment of Neuropsychological Status (RBANS) and some subscales of the MATRICS Consensus Cognitive Battery (MCCB). The secondary outcomes are other behavioral measures, variations in brain imaging, and serum levels of brain-derived neurotrophic factor (BDNF). All outcomes will be measured at baseline, post-treatment, and 3-month follow-up, except for brain imaging and BDNF levels, which will be measured at baseline and post-treatment only.

**Discussion:**

If tDCS combined with WMT results in significant improvements and prolonged effects on working memory, this method could be considered as a first-line clinical treatment for schizophrenia. Moreover, these results could provide evidence-based support for the development of other approaches to improve cognitive function in patients with schizophrenia, especially by enhancing WMT effects.

**Trial registration:**

Chictr.org.cn; ChiCTR2200063844. Registered on September 19, 2022.

**Supplementary Information:**

The online version contains supplementary material available at 10.1186/s13063-022-06776-x.

## Background

Schizophrenia is one of the most common severe mental disorders, and its etiology remains unknown. Cognitive deficits (CD), one of the core symptoms of schizophrenia, affect attention, memory, executive function, and social cognition. Among them, working memory deficits are one of the core and most characteristic of schizophrenia. These deficits are closely related to the social function, daily living ability, and academic performance of patients [1, 2].

Studies have confirmed that central control components play a fundamental role in the working memory system, which is primarily related to dorsolateral prefrontal cortex (DLPFC) function [3], but also involves the basal ganglia and hippocampus [4, 5]. An abnormal functional connection between the prefrontal lobe and other working memory-relevant areas has been hypothesized to underlie working memory deficits in schizophrenia [6, 7]. In addition, the level of brain-derived neurotrophic factor (BDNF), a protein critical for neural plasticity and synaptic signaling, was found to be decreased and closely related to impaired cognitive function in schizophrenic individuals, indicating its critical role in the pathological mechanism of schizophrenia [8–14].

Many studies have shown that repeated intensive and targeted cognitive training, such as cognitive remediation therapy (CRT), significantly improves cognitive deficits [15, 16]. Although several studies have shown that neuropsychological training methods such as CRT can significantly improve the cognitive function of patients with schizophrenia, the overall effect value is around 0.4, which is of moderate level. In particular, for working memory, the effect value was approximately 0.35. A recent systematic review showed that the overall effect of cognitive training was 0.38 and the effect on working memory was 0.29 [17]. Therefore, there is an urgent need to improve cognitive training, especially to increase the effect on working memory.

Studies have shown that cognitive training and sports rehabilitation can improve behaviors, and result in changes in the functioning and functional connections of the trained and related areas. In addition, these changes in brain plasticity are closely related to the persistence of the training effects [18]. The greater the neuroplasticity, the better the training effect and the longer the duration. Therefore, improving the training effect by altering neuroplasticity may be a promising direction for working memory treatment (WMT) [2]. Transcranial direct current stimulation (tDCS) is a safe electrophysiological method for improving neuroplasticity. Short-term tDCS can cause changes in local brain electrical activity and affect cognitive ability. Long-term repeated tDCS by long-term potentiation (LTP) or long-term depression (LTD) leads to continuous changes in the function or functional connectivity of local or related brain regions, ultimately resulting in changes in brain structure and function. One study suggested that a single tDCS (1–2 mA) can temporarily improve executive functions and working memory, as well as learning and memory capabilities in non-affected subjects. After stimulation with a current of 1.5 mA for 20 min, the excitability of the motor cortex was increased by 150% compared with baseline, and the effect lasted for 90 min [19]. A study in elderly subjects found that 10 min tDCS anode stimulation of the left inferior frontal gyrus significantly reversed aging-induced cognitive impairment, reduced bilateral hyperactivity in the prefrontal cortex and anterior cingulate gyrus, diminished wedge-shaped brain activation, and normalized the resting brain function connection mode [20]. A functional magnetic resonance imaging (fMRI) study of patients with schizophrenia found that tDCS stimulation of the left frontal lobe for 30 min resulted in significantly improved working memory performance 24 h post-treatment, a finding that was positively correlated with increased activation in the medial frontal cortex below the anode [21]. A study on tDCS combined with cognitive training found that the latter significantly improved cortical neuroplasticity [22]. Another study of healthy elderly subjects also found that bilateral tDCS of the prefrontal lobe combined with computer cognitive training significantly improved working memory performance. This study showed that during computer cognitive training, tDCS-induced bilateral changes in prefrontal excitability may improve age-related cognitive decline in elderly subjects [23]. These findings suggest that a combination of tDCS and working memory training may be a promising treatment to improve the working memory ability of patients with schizophrenia.

Therefore, we hypothesized that the training approach combining “preheating” (tDCS, which changes the activity of working memory-related brain regions) and “ironing” (WMT) will result in an enhanced effect. To date, no relevant studies have investigated whether or how this combined intervention improves cognitive function, especially working memory, in patients with schizophrenia.

This trial is the first to test the efficacy of the combination of tDCS and WMT on working memory and other cognitive functions in schizophrenia using a double-blind, randomized, controlled design. Numerous neuropsychological and neuroimaging assessments, as well as serum assays, will be performed to better evaluate the efficacy of this intervention. We hypothesized that this study will provide evidence for the greater improvement in cognitive deficits afforded by tDCS combined with WMT, especially for working memory deficits, while elucidating the neuroplastic changes that occur through this combined approach.

## Methods

### Study design

This is a double-center, double-blinded, randomized, controlled intervention trial. This study was registered under Chictr.org.cn (ChiCTR2200063844). This study will be conducted from 2022 to 2024 and reported in accordance with both the Consolidated Standards of Reporting Trials (CONSORT) statement and the CONSORT statement for non-pharmaceutical interventions [24, 25].

The primary objective of this trial is to investigate the facilitation (enhancement) effect of tDCS-induced neuroplasticity changes on the direct and migratory effects of WMT on cognitive function in schizophrenia. The secondary goals are to investigate the effects of tDCS and WMT on the brain plasticity and biological markers of patients with schizophrenia, and to examine the facilitation effect of tDCS on WMT-induced changes in brain plasticity and biological markers.

### Participants

**Fig. 1:**
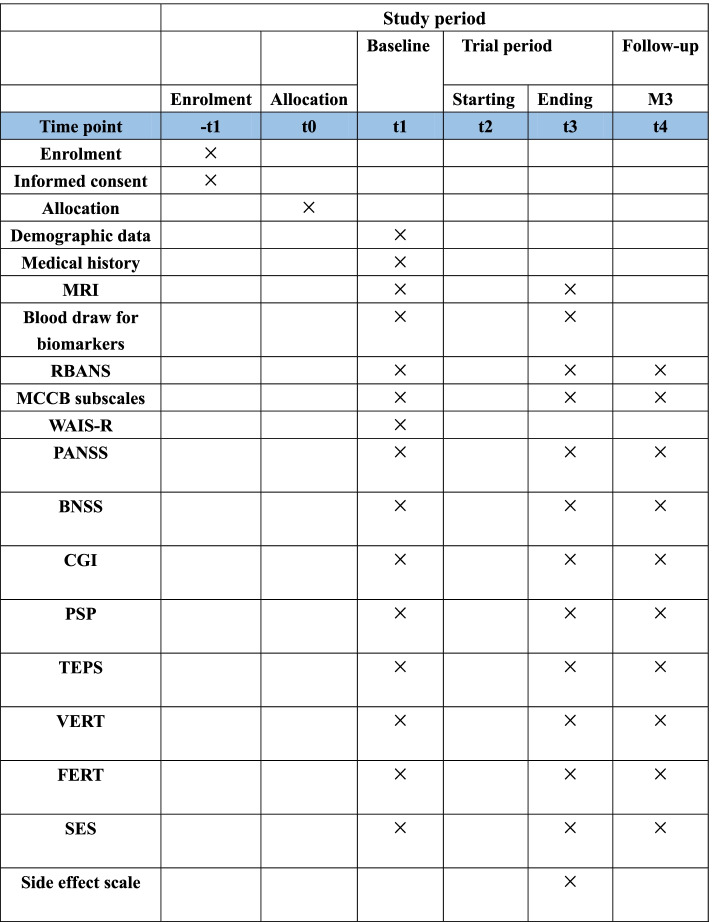
SPIRIT figure showing schedule of enrollment, intervention, and assessments of the study. Repeatable Battery for the Assessment of Neuropsychological Status (RBANS), MATRICS Consensus Cognitive Battery (MCCB), Wechsler Adult Intelligence Test Brief Version (WAIS-R), Positive and Negative Syndrome Scale (PANSS), Brief Negative Symptom Scale (BNSS), the Clinical Global Impression (CGI), Personal and Social Performance Scale (PSP), Temporal Experience of Pleasure Scale (TEPS), vocal emotion recognition test (VERT), facial emotion recognition test (FERT), Self-Esteem Scale (SES)

This study has been approved by the Ethics Committees of Beijing Huilongguan Hospital and Chongqing Three Gorges Hospital Pinghu Branch and conformed to the Declaration of Helsinki (version 2004). The Standard Protocol Items: Recommendations for Interventional Trials (SPIRIT) checklist is detailed in an additional document available upon request, and the schedule of this study is presented in Fig. 1. All patients will sign a written consent to participate in the study after understanding its purpose and procedures.

A total of 120 patients with schizophrenia will be recruited from Beijing Huilongguan Hospital and Pinghu Branch of Chongqing Three Gorges Hospital, according to the following inclusion criteria: diagnosis of schizophrenia according to the Diagnostic and Statistical Manual of Mental Disorders, 5th edition (DSM-V); age between 18 and 60 years; full education of at least 6 years (we ensure that the participants are able to read and understand the task instructions); single score on the Positive and Negative Syndrome Scale (PANSS) < 5 or total PANSS score < 22; evidence of cognitive impairment (the length of number backwards ≤ 6); and right-handedness (Edinburgh Handedness Assessment Scale). The exclusion criteria are as follows: history of an organic brain disorder or other severe organic disorder; diagnosis of substance abuse, as defined by the DSM-V; hearing or visual perception disorders; severe physical diseases or side effects of drugs; inability to carry out cognitive training; severe recession or impulsive excitement; lack of cooperation; pregnancy or lactation; and presence of foreign metal objects or cardiac pacemakers in the body that could prevent MRI scanning. Inclusion and exclusion criteria will be assessed using an electronic case report form (eCRF). Dropping criteria include failure to complete the training ten times in total and unable to continue to participate in the study due to disease relapse or worsening.

### Sample size

The sample size was estimated based on the previous study reporting an effect size of 0.25 for observing the effect of CCRT on cognition [26]. With an alpha error estimate of 0.05 and power (1-β) of 0.90, 4 groups and 3 repeat measurements with a correlation among repetitive measures of 0.5 and nonsphericity correction of 1, a minimum sample size of 52 was required using G*Power 3.1.9.2 [27]. Accounting for the possibility of dropouts, the study adopts the block randomization to divide into groups with 8 patients in each block. The actual sample size of each group was 30 cases. Combining the clinical situations of each center, the Beijing and Chongqing centers were set to provide 80 and 40 patients, respectively.

### Randomization and blinding

Patients will be randomly divided at a ratio of 1:1:1:1 and assigned to one of the following four groups: study group (tDCS + WMT), WMT control group (tDCS + simple response training, SRT), tDCS control group (sham tDCS + WMT), and placebo control (sham tDCS + SRT). Block randomization will be performed in R (The R Foundation, Vienna, Austria) and carried out in 15 blocks of 8 participants. Each patient will be randomly assigned to the baseline group. To eliminate measurement bias, no member of the trial knew which group each patient will be assigned to, except for the caregivers who performed the tDCS treatment. Blinding will be resolved only when urgent treatment is required. Thus, the participant will be managed as off-trial.

### Group interventions

#### *The study group (tDCS* + *WMT)*

Based on previous findings [28, 29], we will apply tDCS at 2 mA for 20 min each, with two stimulations per day separated by 3–4 h. Stimulation will be performed 5 days per week for two consecutive weeks, for a total of 20 stimulations.

We will administer tDCS using a battery-driven stimulator (EM8060, Wuhan YiMai Technology Co., Ltd., Hefei City, China) in Beijing center and a-tDCS (ActivaDose® II, ActivaTeKTM Inc., Gilroy, CA) in Chongqing center. A rectangular anodal electrode will be attached to the left DLPFC (F3 position, according to the International 10/20 system), and a rectangular cathode will be attached to the orbitofrontal cortex. The stimulation area will be wet with saline before treatment.

**Fig. 2:**
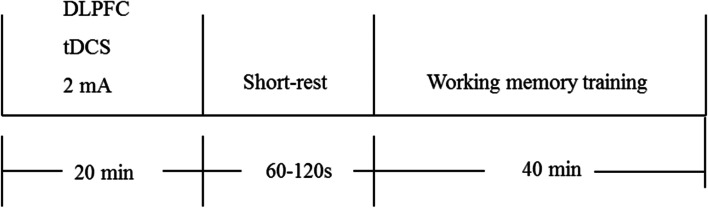
The tDCS + WMT group

Each 20-min tDCS period will be immediately followed by a 1–2-min rest and 40 min of WMT (Fig. 2). The WMT tool is a computer training system based on theoretical models of working memory and cognitive processing. It was self-developed and validated in preclinical research [30]. This training employs the most typical working memory paradigm; that is, subjects try to memorize a series of continuous items (including numbers, words, and pictures) of random (unfixed) length, and judge the order of the 2–4 items presented at the end. Ten WMT sessions will be performed per week for two weeks, for a total of 20 WMT sessions.

#### *The WMT control group (tDCS* + *SRT)*

**Fig. 3:**
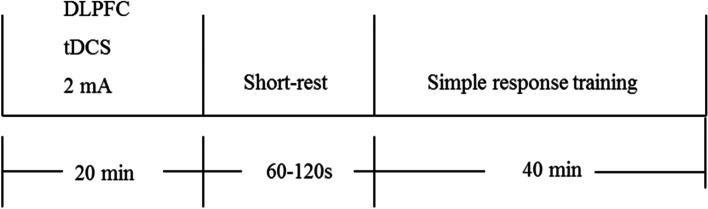
The tDCS + SRT group

The tDCS stimulation protocol is the same as that described in the previous section. Subsequently, SRT will be performed (Fig. 3). SRT requires no working memory processing; thus, it can be used as a comparison condition for WMT. During SRT, the patient must press a key (space) when a specific shape (such as a red triangle) is presented among many sequentially presented geometric figures. To avoid the influence of the individual items on the results, the items of the SRT are the same as those used in the WMT training,

#### *The tDCS control group (sham tDCS* + *WMT)*

**Fig. 4:**
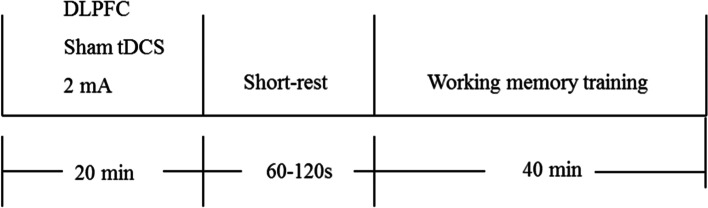
The sham tDCS + WMT group

During each treatment period, the patient will receive 20-min sham tDCS. The stimulation position and parameters, as well as the electrode placement, are identical to those utilized for the tDCS protocol. Electrical stimulation will be administered for only 40 s, and subsequently, its intensity gradually decreases to 0. After a short interval (1–2 min), the patients will undergo WMT with the same training method and duration as the study group (Fig. 4).

#### *The placebo control group (sham tDCS* + *SRT)*

**Fig. 5:**
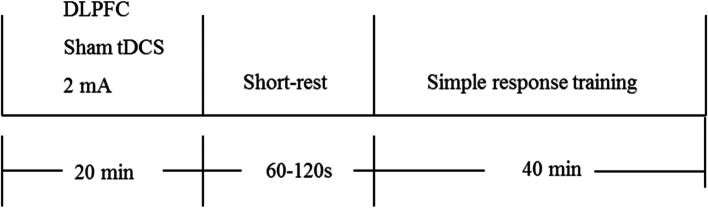
The sham tDCS + SRT group

During each treatment period, the patients will receive 20-min sham tDCS. After a short interval (1–2 min), simple reaction training will be performed (Fig. 5).

### Outcome measures and assessment

The primary outcome measure is cognitive function measured using Repeatable Battery for the Assessment of Neuropsychological Status (RBANS) and some subscales of the MATRICS Consensus Cognitive Battery (MCCB). Secondary outcomes are behavioral changes, modifications in brain structural and functional plasticity as measured by MRI, and serum levels of BDNF. All participants from both hospitals will be assessed by neuropsychologists with considerable training.

### Data collection and follow-up

Investigators will visit the participants before the beginning of the trial (baseline), at the end of the trial, and 3 months after the last treatment. Calls will be required to keep in touch with the patients and remind them of their next interview to avoid loss to follow-up.

**Fig. 6:**
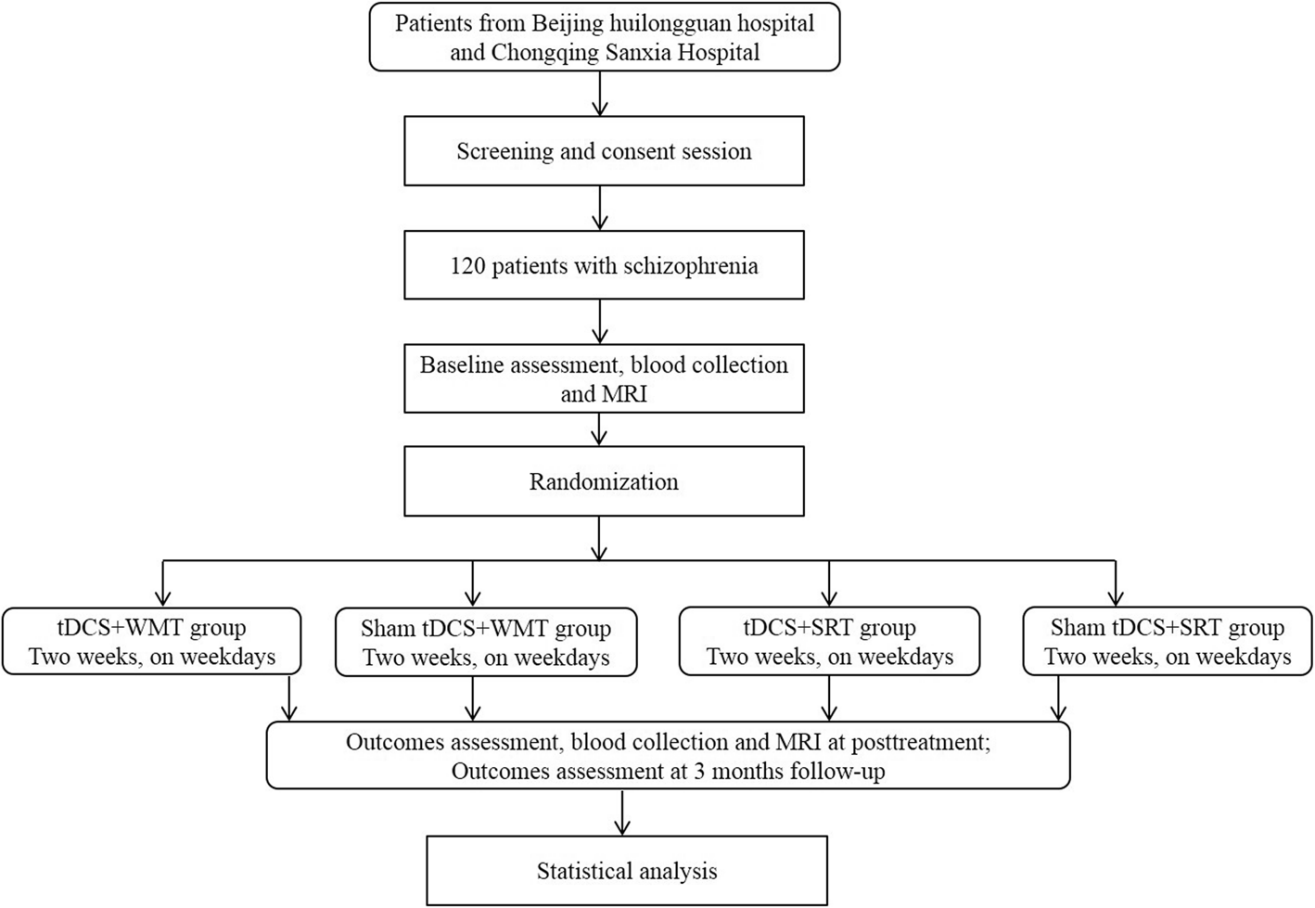
The treatment flowchart

Baseline demographic data (age, sex, education, occupation, etc.), smoking status, current medication, concomitant medications, detailed physical and neurological findings, functional and structural brain MRI, the serum BDNF assay, and neuropsychological data will be collected. A follow-up assessment will be scheduled post-treatment and three months after the last cognitive training. Neuropsychological assessments will be performed post-treatment and at the three-month follow-up. Functional and structural brain MRI and the serum BDNF assay will be also performed post-treatment (Fig. 6).

### Neuropsychological assessments

Cognitive function will be measured with RBANS and some subscales of the MCCB and the Wechsler Adult Intelligence Test Brief Version (WAIS-R). The primary evaluation indicators of a training effect on working memory are the spatial span and digital sequence subscales in the MCCB and the RBANS. Evaluation indicators of the migration effect are reasoning and problem solving ability evaluated by the maze test and social cognition ability determined by the vocal emotion recognition test (VERT) and facial emotion recognition test (FERT).

Clinical symptoms will be evaluated using the Chinese version of the PANSS, Brief Negative Symptom Scale (BNSS), and Clinical Global Impression (CGI). These will be assessed by four clinically trained senior physicians at each center.

Social function will be determined with the Personal and Social Performance Scale (PSP). Other scales employed are the Temporal Experience of Pleasure Scale (TEPS), Self-Esteem Scale (SES), and side effects scale*.*

The results from the above mentioned neuropsychological tests will be blindly assessed by raters who are unaware of the treatment the patients are receiving. Except for the WAIS-R, which will be assessed only once at baseline, the other scales will be assessed at three time points (baseline, post-treatment, and 3-month follow-up).

### MRI protocol

Brain MRI will be performed at Beijing Huilongguan Hospital. Due to limited experimental conditions, patients of Chongqing Three Gorges Hospital will not undergo MRI.

Patients will undergo a brain MRI at baseline and after treatment. MRI will be performed using an optimized protocol with a 3.0-T MRI scanner (Siemens Healthcare, Prisma, Germany). The fMRI data will be analyzed using statistical parametric mapping (SPM) (UCL Queen Square Institute of Neurology, London, UK). Changes in brain activation and connectivity between baseline and post-treatment will be compared between the four groups.

### Blood sampling

Serum samples will be obtained to analyze BDNF levels at baseline and post-treatment. Peripheral venous blood (10 mL) will be collected from 7:00 am to 9:00 am in a climate-controlled room following overnight fasting. Blood samples will be centrifuged at 4000 g for 15 min at 4 °C. Serum and plasma will be separated and stored at − 80 °C. Serum levels of BDNF will be measured using enzyme-linked immunosorbent assay (ELISA) kits (R&D Systems, Minneapolis, MN, USA).

All samples will be measured by the same technician who is blinded to the patient treatment status. All samples will be evaluated in duplicate and averaged.

### Data management and monitoring

The data will be kept strictly confidential and stored in the Alibaba Cloud through eCRF. Every member of the team will create a personal account for data entry and modification. A correction function will be run in the background of eCRF to prevent errors. The data sets and informed consent forms analyzed during the current study are available from the respective authors upon reasonable request.

A project management team is created to supervise the overall work of the two centers. The trial review team consists of two staff members from the review department and is responsible for a regular review of progress, authenticity, and safety every 6 weeks.

The intervention may produce mild adverse events such as headaches and irritability, which can be improved by providing patients with rest or interval stimulation. If patients showed intolerance, they could opt out of the trial. This situation should be reported to the supervisory department. Trial audit teams, independent data monitoring, and ethics committees must review the conduct throughout the trial.

### Statistical analysis

Analyses will be performed according to the intent-to-treat (ITT) principle. Descriptive analysis will be performed on the data collected during each assessment at baseline, post-treatment, and the 3-month follow-up. The median and range describe continuous variables, and the frequencies and percentages describe qualitative variables.

Statistical significance will be set at *P* < 0.05, and corrected for multiple comparisons, when needed. To assess our primary endpoint criterion, that is, changes in cognitive performance, the outcomes at the three time points will be compared using a repeated-measures analysis of variance (ANOVA) with time (baseline, post-treatment, and 3-month follow-up) and group (tDCS + WMT, tDCS + SRT, sham tDCS + WMT, and sham tDCS + SRT) as factors. Further comparisons between the two groups can show the simple tDCS effect, simple WMT effect, and promoting effect of tDCS on WMT. Sensitivity analyses will be used to explore the effect of missing data due to loss to follow-up and dropout.

In cases where the data do not satisfy the assumption of normal distribution, the non-parametric Mann–Whitney *U* test will be used to compare the performance improvement between the four groups. SPM will be used to analyze imaging data to detect changes in brain function caused by cognitive training.

## Discussion

As far as we know, working memory deficits are one of the core features of cognitive impairment and are closely related to social function in patients with schizophrenia. Although current WMT methods can improve cognitive performance, their overall and transfer effects are relatively low. More effective methods are urgently required to enhance the effects of WMT. This study is the first to combine tDCS stimulation with WMT to enhance the effect of WMT, altering the neuroplasticity of related brain regions in patients with schizophrenia.

This study has several strengths. As a well-documented effective method for altering brain plasticity, tDCS stimulation is used to modulate the functional state of brain regions prior to WMT, and functional MRI is used to explore the effects of tDCS on brain functional plasticity in patients with schizophrenia. In addition, a key issue for WMT mechanism studies is determining whether tDCS stimulation first activates local neuronal activity, subsequently enabling WMT to produce more pronounced changes in brain function. To determine this, we compare the tDCS + SRT and sham tDCS + WMT groups to explore whether the tDCS combined with the WMT approach would be superior to either in altering neuroplasticity (function and structure). Our findings help to confirm our hypothesis that tDCS can facilitate the clinical effect of WMT on cognitive improvement in schizophrenia and provide a scientific basis for exploring ways to enhance brain neuroplasticity and improve its function. Another strength of this trial is the exploration of the transfer effect, as our data will determine whether training, such as WMT, can improve untrained cognitive ability, including logical reasoning. The transfer effects of WMT on healthy individuals have already been confirmed, but few studies have investigated whether such effects also occur in schizophrenia. This study will fill this gap in knowledge.

This study has some limitations that must be addressed. First, the enrolled participants are not fully representative of a patient population with other comorbidities and considerable treatment heterogeneity. Therefore, we chose strict inclusion, exclusion, and dropping criteria to reduce the effects of confounding variables and to render the trial more accurate and realistic. Second, there may be challenges regarding the participants’ reaction to the trial, e.g., if the participant believes they are receiving sham treatment or if the treatment is too uncomfortable or too tiring. It is not clear whether participants will be able to distinguish between the real and sham treatments and the impact this may have on attrition rates.

## Trial status

This is the version of the protocol, dated September 19, 2022. Recruitment for this trial will begin soon and is expected to finish by the end of 2024.

## Supplementary Information

**Additional file 1.** Spirit Checklist.

## Data Availability

The datasets are available from the corresponding author upon request.

## Abbreviations

BDNF: Brain-derived neurotrophic factor
BNSS: Brief Negative Symptom Scale
BVMT: Brief Visuospatial Memory Test
CGI: Clinical Global Impression
CONSORT: Consolidated Standards of Reporting Trials
CPT: Continuous Performance Test
eCRF: Electronic case report form
FERT: Face Emotion Recognition Test
HVLT: Hopkins Verbal Learning and Memory Test
LTP: Long-term potentiation
LTD: Long-term depression
MCCB: MATRICS Consensus Cognitive Battery
MRI: Magnetic resonance imaging
PANSS: Positive and Negative Syndrome Scale
PSP: Personal and Social Performance Scale
SES: Self-Esteem Scale
SPIRIT: Standard Protocol Items Recommendations for Interventional Trials
SQOLS: Schizophrenia Quality of Life Scale
SRT: Simple response training
tDCS: Transcranial direct current stimulation
TEPS: Temporal Experience of Pleasure Scale
VERT: Vocal Emotion Recognition Test
WAIS-R: Wechsler Adult Intelligence Test Brief Version
WMT: Working memory training
